# The CLASS Surgical Site Characteristics in a Clinical Grading Scale and Anterior Segment Optical Coherence Tomography: A One-Year Follow-Up

**DOI:** 10.1155/2018/5909827

**Published:** 2018-05-15

**Authors:** Judyta Jankowska-Szmul, Edward Wylegala

**Affiliations:** Department of Ophthalmology, School of Medicine with the Division of Dentistry in Zabrze, Medical University of Silesia, Katowice, Poland

## Abstract

**Purpose:**

We combined a clinical grading scale and swept source anterior segment OCT to describe the successful and failed CLASS.

**Material and Methods:**

23 patients in the successful group and 17 patients in the failed group were compared in terms of the IBAGS grades and AS-OCT findings at one, three, and twelve months postoperatively.

**Results:**

The majority in the successful group presented shallow blebs (91%, 57%, and 52% at 1M, 3M, and 12M, resp.). 59% of the failed group presented no bleb (H0 E0) from the early postoperative period with the rate increasing to 88% at 3M and 100% at 12M. The scleral lake was detected in all the successful patients. The successful group showed significantly higher rates of TDM integrity (*P* < 0.001), IF (*P* < 0.001), and SCF (*P* < 0.05), but there were no significant differences in the rates of microcysts between the groups (*P* > 0.05). We found a significant decrease in the SL anteroposterior extent (*P*=0.003) and SL height (*P* < 0.001) over time, with no significant correlation between the above parameters and IOP.

**Conclusions:**

The subconjunctival bleb may be a sign of the successful CLASS when it matches the AS-OCT findings of TDM integrity, maintained scleral lake, and intrascleral fluid. A validated OCT pixel intensity measurement is required to evaluate the bleb reflectivity.

## 1. Introduction

The introduction of the Moorfields Safer Surgery System has improved the surgical outcomes and safety profile of trabeculectomy [1, 2]. At the same time, the continuous search for safe glaucoma surgery motivates surgeons to revise nonpenetrating procedures. CO_2_ laser-assisted sclerectomy surgery (CLASS) is a recent modification of nonpenetrating deep sclerectomy (NPDS) and has been implemented to overcome technical difficulties of the manual technique [3, 4]. During the sclerectomy procedure, the CO_2_ laser energy provides accurate ablation of dry tissues and is absorbed by fluid, which means that the aqueous humour percolating through the intact trabeculo-Descemet's membrane (TDM) intrinsically prevents excessive ablation. Such an approach reduces potential risk of the intraoperative TDM perforation and the whole procedure becomes less dependent on the surgeon's skills [3, 5]. CLASS outcomes, in terms of intraocular pressure-lowering potential, have already been reported as comparable to classic NPDS over a period of up to two-year follow-up [6–8]. To the best of our knowledge, no scientific data on surgical site characteristics following the successful or failed CLASS have been published so far. The information on external and internal bleb characteristics in trabeculectomy and NPDS has been found useful to understand the wound healing process and the reasons for different surgical outcomes. It has been acknowledged that the long term success of glaucoma filtration surgery depends on adequate formation and maintenance of the filtering bleb. Standardized classifications of the bleb morphology have been proposed to establish uniform assessment of external bleb features following trabeculectomy. Two most commonly used clinical grading systems, which have demonstrated high interobserver agreement and clinical reproducibility, are the Indiana Bleb Appearance Grading Scale (IBAGS) and the Moorfields Bleb Grading System (MBGS) [9–11]. The IBAGS evaluates the bleb height, extent, vascularity, and leakage (Seidel test), which are the primary features bearing the greatest significance in the bleb functionality and prognosis for successful intraocular pressure (IOP) control [9]. IBAGS has been adopted to evaluate the bleb morphology in NPDS, and it has been reported that the bleb formation contributes to outcomes of the procedure [12, 13]. However, as the subconjunctival bleb develops only in up to 50% of successful NPDS patients, the external bleb assessment may not correlate well with the IOP [13]. The mechanism of IOP reduction in the NPDS is complex and involves subconjunctival bleb, intrascleral bleb, suprachoroidal outflow, and episcleral vein outflow of aqueous from Schlemm's canal [14, 15]. Formation and long-term maintenance of an intrascleral space, also called an intrascleral or scleral lake, are thought to be important in facilitating aqueous humour drainage in NPDS [16]. A number of adjunctive materials has been implanted in the scleral resection bed in order to maintain a patent scleral lake, such as collagen implants [17], reticulated hyaluronic acid [18], and nonresorbable acrylic implants [19, 20]. As the intrascleral structures and residual tissue morphology in NPDS cannot be evaluated by the slit-lamp examination, the use of other examination techniques has been proposed. Previous studies have reported the use of ultrasound biomicroscopy (UBM) [21, 22], but more recently, anterior segment optical coherence tomography (AS-OCT) [23–26] and in vivo confocal microscopy (IVCM) [27–29] have been adopted for the bleb analysis. Compared with the UBM, the AS-OCT offers not only superior image resolution and reproducibility, but as a noncontact imaging, it neither affects the bleb morphology nor carries the risk of infection [21, 24, 26]. Various AS-OCT and IVCM parameters have been reported as associated with the bleb function after trabeculectomy, including low intrableb reflectivity and extensive hyporeflective areas [25, 30, 31], the presence of an aqueous outflow channel at the scleral flap [24, 32], a large number of microcysts [28, 30], a large subconjunctival hyporeflective space with multiloculated fluid collections [24] and loosely arranged subepithelial connective tissue [33]. A bleb cavity formed superiorly to the scleral flap has been described for trabeculectomy, whereas a fluid reservoir formed inferiorly to the scleral flap has been reported in NPDS [34]. In terms of correlation between the IOP and the scleral bleb measurements, findings for the NPDS are contradictory. Some authors found an inverse correlation between the scleral lake height, anteroposterior extent, or volume and the postoperative IOP [12, 13, 35], while the others concluded that the lake size does not correlate with the degree of IOP reduction [36, 37].

In the following study, we aim to provide a complex report on surgical site characteristics in CLASS. We combine a clinical grading scale and swept source anterior segment optical coherence tomography to describe the external and scleral bleb findings in the successful and failed procedure. We also investigate into the correlation between scleral lake measurements and postoperative IOP over the period of 12 months following the successful surgery.

## 2. Materials and Methods

### 2.1. Study Design

This was a non-controlled prospective study, including patients who underwent CLASS as a primary glaucoma surgery for primary open angle glaucoma (POAG) or open angle glaucoma secondary to pseudoexfoliation syndrome (exfoliative glaucoma, XFG) in our hospital. The study was approved by the Local Ethics Committee (KNW/0022/KB1/132/16) and conducted according to the tenets of the Declaration of Helsinki. Informed written consent was obtained from all the patients. Inclusion criteria were as follows: adult patients (aged ≥18), medically uncontrolled POAG or XFG, open angles in gonioscopy (grade 3 or 4 in four quadrants, according to the Shaffer classification), pseudophakia and a history of uneventful cataract surgery performed >6 months prior to listing for the glaucoma surgery. Patients with a history of any other eye surgery were not included.

The patients underwent a baseline examination within 2 days before the surgery and were followed up for 12 months postoperatively. At postoperative month 1 (1M), month 3 (3M), and month 12 (12M), we recorded IOP as an average of three consecutive measurements with a calibrated Goldman applanation tonometer (Haag-Streit AG, Koeniz, Switzerland), a number of glaucoma medications, bleb slit-lamp photographs, and bleb AS-OCT scans. The photos and the scans of the superior quadrant were acquired in downgaze, with the upper eyelid lifted up to expose the bleb without pressing on the eye surface. “Complete success” was defined as IOP measured at each follow-up visit ranging between 10 mmHg and 18 mmHg and reduced by at least 30% from the baseline without glaucoma medications. “Qualified success” was defined as IOP measurements meeting the above criteria with or without glaucoma medications. IOP <10 mmHg or >18 mmHg despite the medications, reduction of less than 30% from the baseline, reoperation for glaucoma within 12 months, or loss of light perception was classified as failure.

#### 2.1.1. Indiana Bleb Grading Scale (IBAGS)

**Figure 1:**
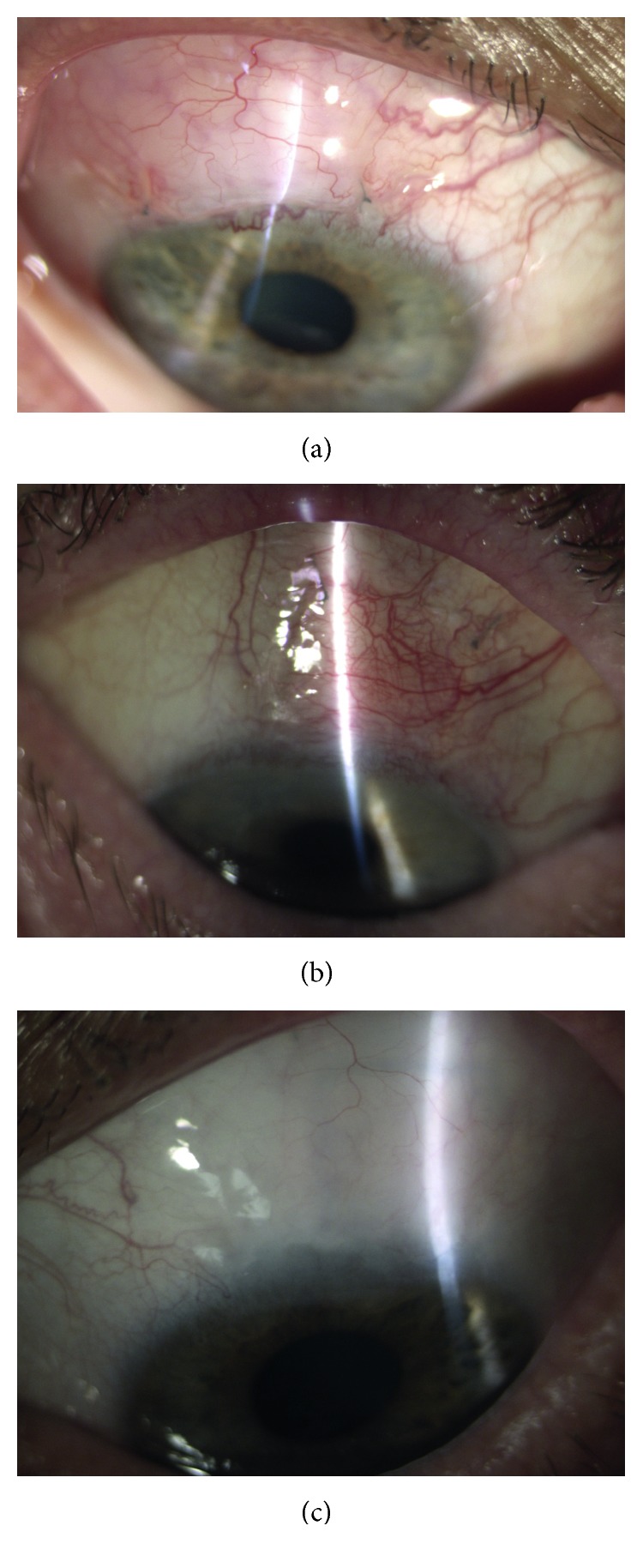
(a)–(c) Representative bleb photographs: examples of the IBAGS grades. (a) The successful CLASS at 1M: H2 E2 V2 S0. (b) The failed CLASS at 3M: H0 E0 V3 S0. (c) The successful CLASS at 12M: H1 E2 V2 S0.

The external bleb morphology in the photographs was scored according to the IBAGS. The blebs were assessed in terms of their four features: height (H0–3), extent (E0–3), vascularity (V0–4), and leakage (S0–2) [9] (Figures 1(a)–1(c)).

#### 2.1.2. Anterior Segment OCT

**Figure 2:**
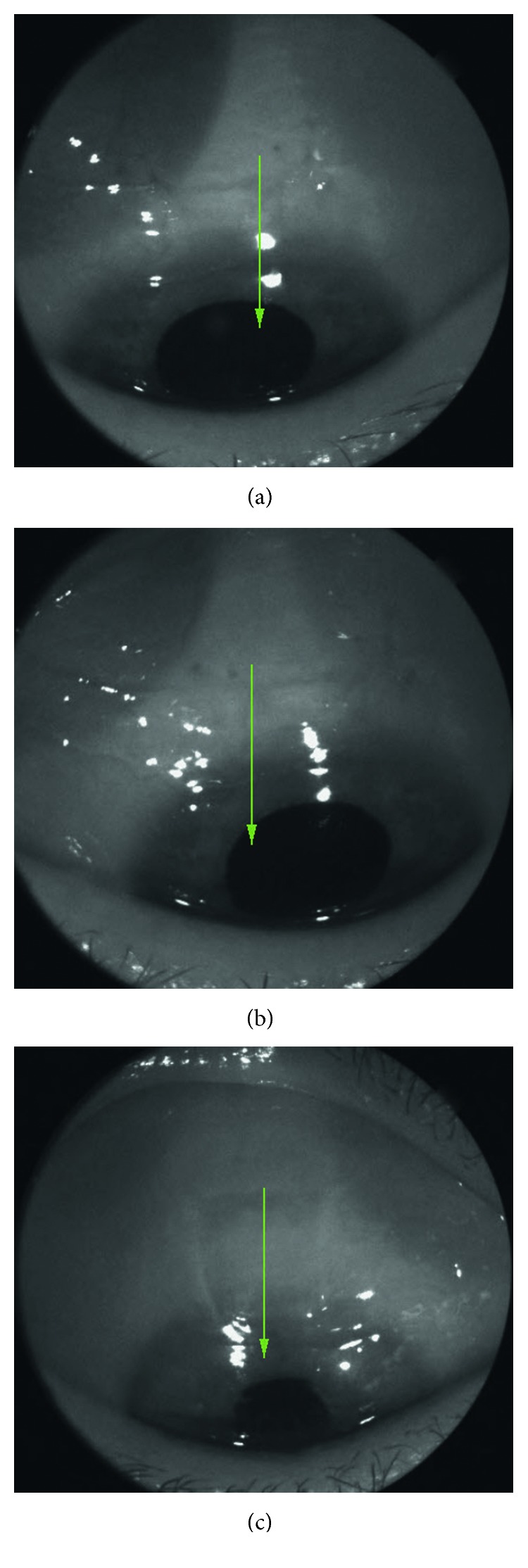
(a)–(c) DRI OCT Triton, “Anterior scan 6 mm.” Anterior segment photographs presenting the location of cross-sectional images obtained vertically over the medial and the marginal aspects of the scleral flap, as indicated by the green arrow. (a) Left margin; (b) right margin; (c) medial zone.

The images were acquired with a commercially available swept source OCT machine (DRI OCT Triton, Topcon, Inc., Tokyo, Japan), using the anterior attachment. The device performs 100,000 A-scan image acquisitions per second with a wavelength of 1050 nm. The protocol consisted of a series of nine cross-sectional images (“anterior scan 6 mm”) per patient. The scans were acquired vertically over the medial and marginal aspects of the scleral flap (Figures 2(a)–2(c)), as follows: left margin (three scans taken 0–0.5 mm from the left scleral flap edge, perpendicular to the limbus), right margin (three scans taken 0–0.5 mm form the right scleral flap edge, perpendicular to the limbus), and medial zone (three scans taken in the middle of the scleral flap, i.e., 2 mm from the flap edge, perpendicular to the limbus), as indicated by the green arrow.

**Figure 3:**
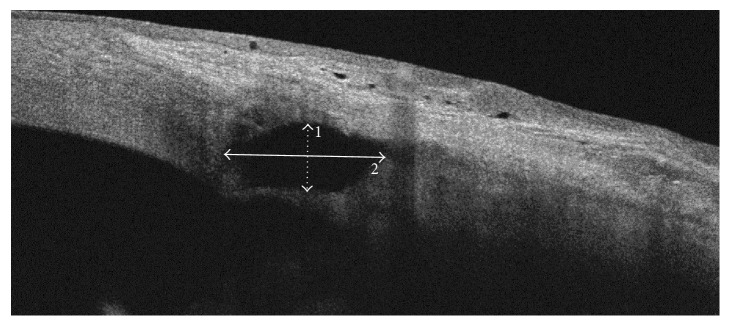
DRI OCT Triton, anterior scan 6 mm. A representative scan showing the quantitative assessment. 1: scleral lake height (dot line). 2: scleral lake anteroposterior extent (solid line).

Quantitative assessment included scleral lake height and scleral lake anteroposterior extent measurements (SL height and SL a-p extent). They were taken with the OCT machine calliper tool in a single scan and then calculated as an average of the maximal measurements from the nine scans (Figure 3).

**Figure 4:**
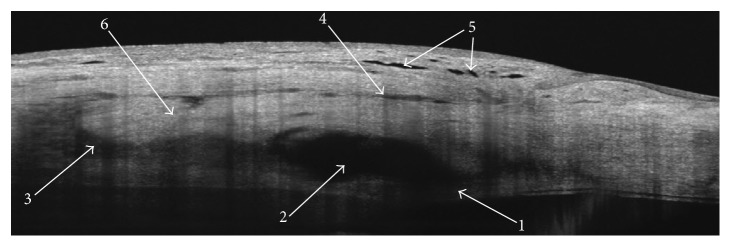
DRI OCT Triton, anterior scan 6 mm. A representative scan showing the qualitative assessment. Hyperreflective strand of the intact trabeculo-Descemet's membrane separates a well-defined hyporeflective scleral lake. Hyporeflective trace of the intrascleral fluid communicates with the scleral lake and extends inferiorly and posteriorly to the scleral flap. Hyporeflective pockets of the subconjunctival fluid are identified superior to the scleral flap. Microcysts are recognised as multiple hyporeflective fluid collections within the conjunctiva. Scleral flap is marked to help identify the structures 1–5. 1: trabeculo-Descemet's membrane integrity, 2: scleral lake (SL), 3: intrascleral fluid (IF), 4: subconjunctival fluid collections (SCF) and 5: conjunctival microcysts (microcysts). 6: scleral flap.

Qualitative assessment of each scan included documenting the evidence of five features: trabeculo-Descemet's membrane integrity (TDM integrity), scleral lake (SL), intrascleral fluid (IF), subconjunctival fluid collections (SCF), and conjunctival microcysts (microcysts) (Figure 4).

#### 2.1.3. Quantitative and Qualitative Data Presentation

The “complete success” group and the “failure” group patients were compared in terms of the IBAGS scores and qualitative AS-OCT findings rates at 1M, 3M, and 12M (Tables 1 and 2). In the complete success group, the average scleral lake height and anteroposterior extent (SL height and SL a-p extent) measurements at 1M, 3M, and 12M were compared (Tables 3 and 4). The correlation coefficients between the SL height or SL a-p extent and IOP at 1M, 3M, and 12M were calculated (Table 5).

### 2.2. Surgical Procedures

**Figure 5:**
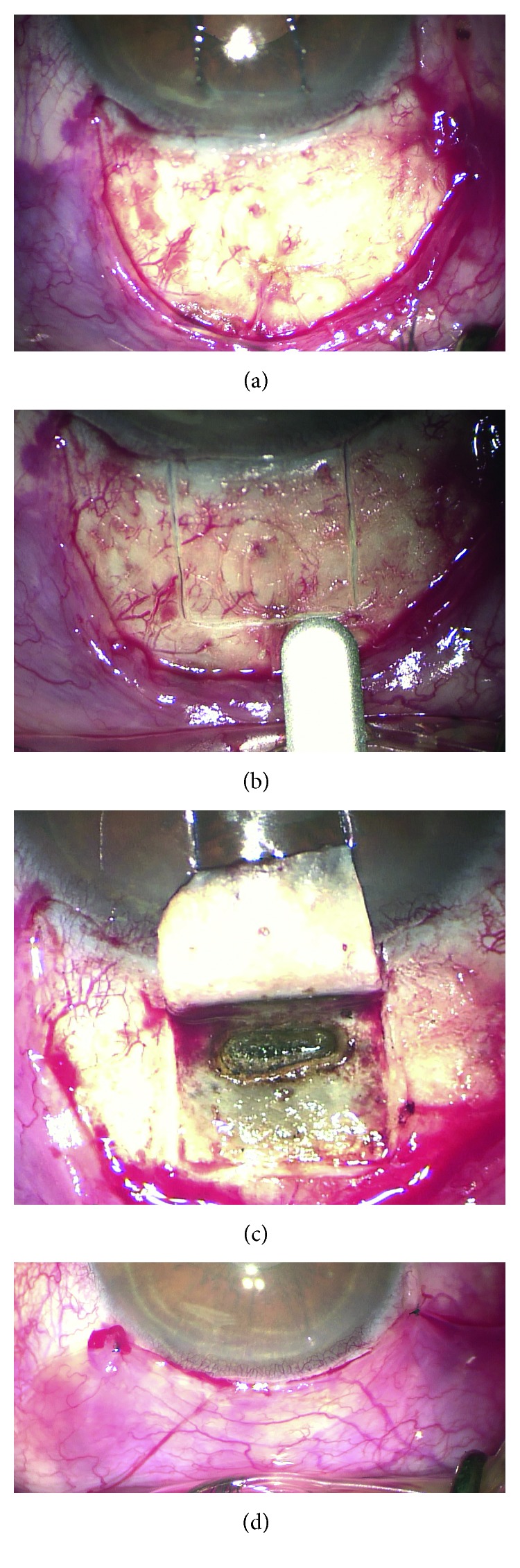
(a)–(d) Intraoperative photographs of CLASS. (a) Peritomy and tenonectomy within the exposed sclera. (b) Lamellar scleral flap dissection with a crescent knife. (c) Progressive CO_2_ laser dissection of the deep sclera and Schlemm's canal towards the trabeculo-Descemet's membrane to achieve aqueous percolation. The laser beam is not visible in the picture. (d) Closure of the scleral flap and conjunctiva, a nice filtering bleb.

CLASS was performed following the technique described by Geffen et al. (2012; 2014), using a commercially available OT-135 CO_2_ laser system (IOPtiMate; IOPtima Ltd, Ramat Gan, Israel), by the same experienced surgeon (EW). The surgery was done under peribulbar anaesthesia (3 : 1 of bupivacaine 0.5% and lidocaine 2%), in the superior quadrant, using the corneal traction suture. The procedure involved conjunctival peritomy, opening and dissection of Tenon's capsule, cauterization of bleeding vessels within the area of the exposed sclera (Figure 5(a)), and dissection of 50% lamellar scleral flap of dimensions 4 mm × 4 mm at the 12 o'clock position using a feather blade (initial horizontal incision) and a crescent blade (flap dissection) (Figure 5(b)). The laser dissection of the deep sclera and Schlemm's canal towards the trabeculo-Descemet's membrane was performed to achieve aqueous humour percolation (Figure 5(c)). The scleral flap edges were approximated with two fixed 8.0 nylon intrascleral sutures, applied at the corners of the flap, and conventional conjunctival closure was performed with two to four 9.0 nylon-interrupted sutures (Figure 5(d)). There was no application of perioperative antimetabolites and no use of intrascleral space–maintaining materials. Postoperative management included routine overnight patching, topical levofloxacin 5 mg/ml, which continued for four weeks, and dexamethasone 1 mg/ml, which tapered down over eight weeks.

### 2.3. Statistical Analysis

Descriptive statistical results were presented as mean ± standard deviation (SD) or median with range. Normality of the data was tested with the Shapiro–Wilk test. Correlation between two quantitative variables was assessed with the Pearson correlation coefficient (if both variables were normally distributed) or with the Spearman rank correlation coefficient (otherwise). Comparison of three or more repeated measurements of the quantitative variable was conducted with the repeated measures ANOVA (if normality assumption was met) or Friedman test (otherwise). In the case of detecting significant differences, post hoc analysis was done (with the paired Student's *t*-test or paired Wilcoxon tests, both with Bonferroni correction). Comparison of qualitative variables was conducted with the chi-squared test (with Yates correction) or Fisher's exact test. A *P* value of less than 0.05 was considered significant. The analysis was performed in R package, version 3.4.2.

## 3. Results

### 3.1. Patient Demographics, Baseline Characteristics, and Success Rates at 12 Months

66 Caucasian patients (66 eyes) were enrolled in the study. The baseline demographic characteristics of the study group are summarized in Table 6.

At the end of the follow-up period of the study, complete success was achieved in 23 (35%) patients, qualified success in 49 (74%) patients, and failure in 17 (26%) patients (Table 7). The analysis was performed for the group of 23 patients with the complete success (group 1: successful) and for the group of 17 patients with the failed procedure (group 2: failed). Seven patients underwent additional glaucoma surgery (trabeculectomy) between three and twelve months following CLASS, so they were excluded from the further analysis, and therefore, the number of patients in the failed group at 12M decreased from 17 to 10.

### 3.2. IBAGS Scores

Through the follow-up period of the study, the majority of patients in the successful group presented shallow blebs (H1 rate decreasing from 91% to 57% and 52% at 1M, 3M, and 12M, resp.) (Table 1). Initially, there was no evidence of flat blebs in this group (0% at 1M), but H0 rates reached 43% at 3M and 48% at 12M. There were significant differences in the bleb height and extent observed in both groups (*P* < 0.05). Both H0 and E0 grades were observed at significantly higher rates in the failed group (59%, 88%, and 100%; *P* < 0.05). In terms of the bleb extent at 1M, 3M, and 12M, the successful patients presented increasing rate of E0 (9%, 43%, and 48%, resp.) and decreasing rate of E2 (83%, 48%, and 43%, resp.). The failed group patients at 1M were graded as E0 (59%) or E1 (41%), with the E0 rate increasing to 88% at 3M and 100% at 12M. In terms of the bleb vasculature, at 1M all the successful patients showed V2 grade, with the rate decreasing to 83% up to 12M. At 3M and 12M, however, there was no significant difference in V2 rates between the groups. None of the successful patients presented with V3, while this grade was given to 5 patients (29%) from the failed group at 1M and 3M. The above five patients underwent trabeculectomy due to CLASS failure between three and twelve months of the follow-up period of the study. One patient in the failed group developed the positive Seidel test at 1M (S1). Figures 1(a)–1(c) show representative bleb photographs of the patients from both groups.

### 3.3. AS-OCT Findings

**Figure 6:**
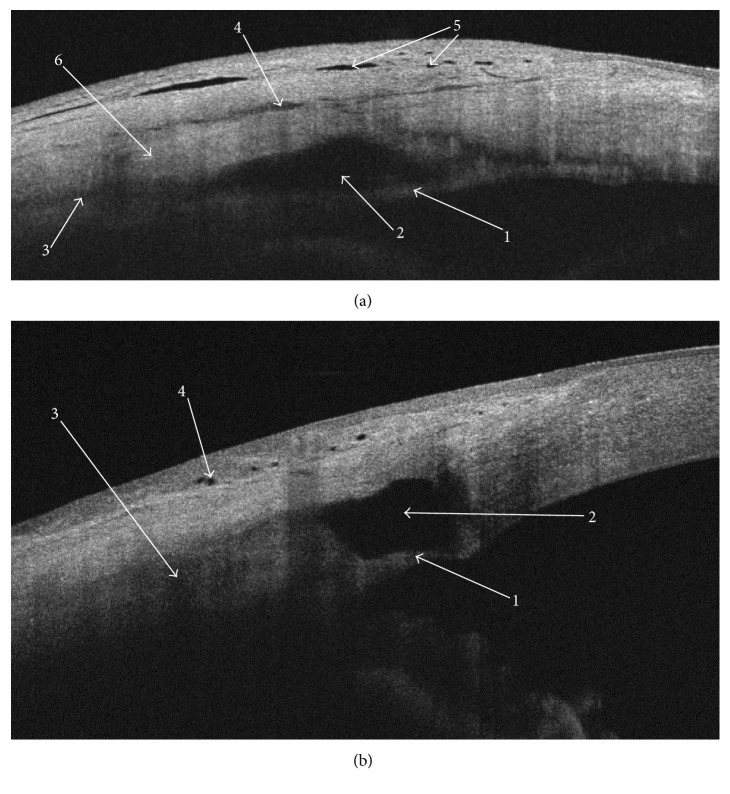
(a, b) Two clinical examples of the successful CLASS at 12M. AS-OCT scans of the medial zones presenting intact trabeculo-Descemet's membrane and well-maintained scleral lake, with the different morphology of sclera. (a) Distinctive loose architecture of scleral and conjunctival tissues with multiple fluid collections. 1: hyperreflective strand of the intact TDM. 2: well-defined hyporeflective scleral lake. 3: hyporeflective trace of IF communicating with the scleral lake and extending inferiorly and posteriorly to the scleral flap. 4: hyporeflective subconjunctival fluid collecting superiorly to the scleral flap. 5: hyporeflective microcysts. 6: scleral flap. (b) A well-defined scleral lake. No evident intrascleral fluid collections elsewhere. 1: TDM. 2: scleral lake. 3: debatable finding of hyporeflective trace of fluid located posteriorly to the scleral flap. 4: microcysts.

**Figure 7:**
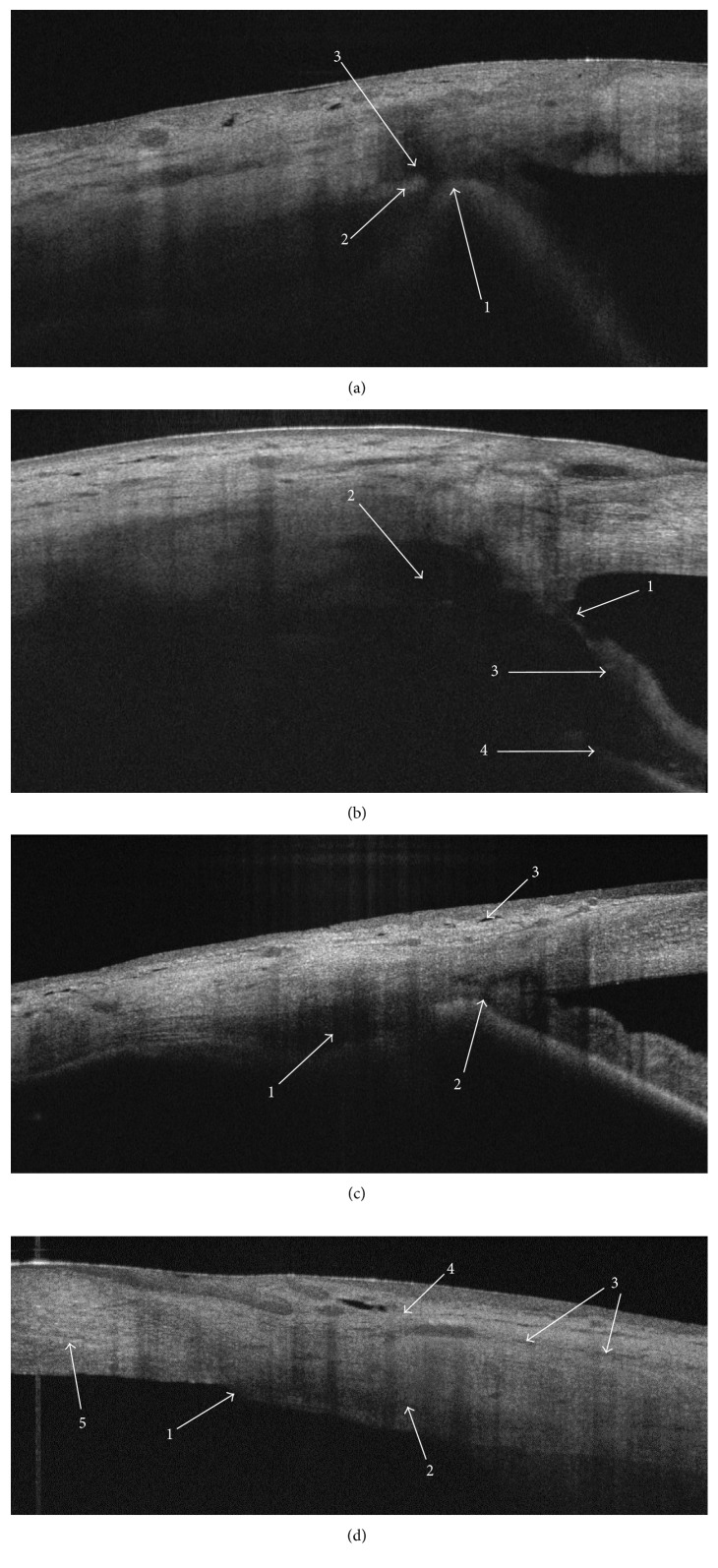
(a)–(d) Four clinical examples of the complicated and failed CLASS. (a) Complicated CLASS at 1M. Spontaneous TDM perforation and iris incarceration, probably subsequent to the intraoperative microperforation. 1: hyperreflective iris pressing on the area of sclerectomy. 2: remnants of the hyperreflective TDM. 3: collapsed scleral lake. (b) Complicated CLASS at 1M. Postoperative anterior uveitis resulting in peripheral anterior synechia adjacent to the area of sclerectomy. 1: synechia obscuring the TDM. 2: scleral lake. 3: iris stroma. 4: pigmented iris epithelium. (c) Failed CLASS at 3M. The hyporeflective space located posteriorly to the iridocorneal angle suggests inaccurate laser ablation. 1: hyporeflective niche in the area of laser applications. 2: intact iridocorneal angle. 3: microcysts. (d) Failed CLASS at 3M due to early fibrosis. No evidence of intrascleral fluid. 1: no TDM and no scleral lake in area of the presumed sclerectomy. 2: hyporeflective trace of the inner margin of the scleral flap. 3: hyporeflective trace of the outer margin of the scleral flap. 4: conjunctiva.

Scleral lake was the only AS-OCT finding detected in all the successful group patients (100%) through the whole follow-up period of the study (Table 2). While the successful group presented significantly higher rates of TDM integrity (*P* < 0.001), IF (*P* < 0.001), and SCF (*P* < 0.05), there was no significant difference in the rates of microcysts found in both groups (*P* > 0.05) (Table 2). Figures 6 and 6 and Figures 7(a)–7(d) show qualitative findings in the cases of successful, complicated, and failed CLASS. In the failed group, AS-OCT revealed inaccurate ablation (two patients), anterior synechia (two patients), iris incarceration (two patients), and complete fibrosis (eight patients) (Figures 7(a)–7(d)).

**Figure 8:**
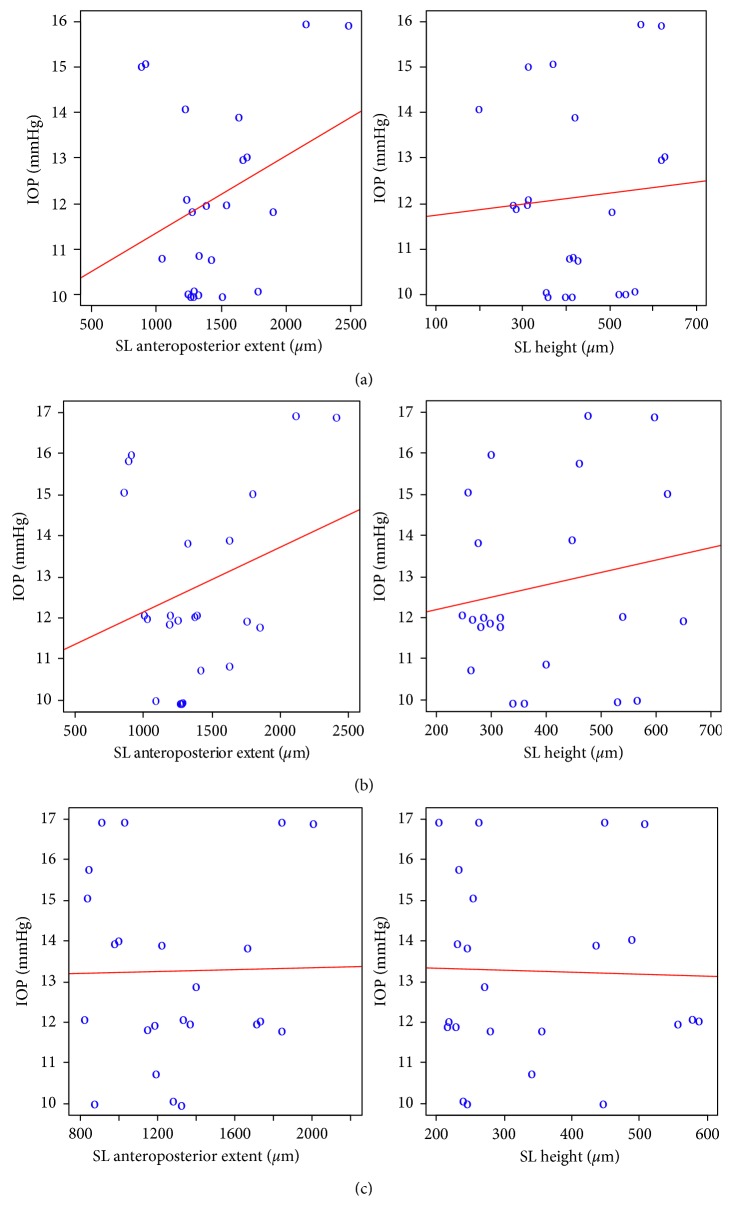
(a)–(c) Scatterplots showing correlations between the AS-OCT scleral lake measurements (SL anteroposterior extent and SL height) and intraocular pressure (IOP) at 1 month (1M), 3 months (3M), and 12 months (12M).

There was a significant decrease in the SL a-p extent (*P*=0.003) and SL height (*P* < 0.001) over time (Tables 3 and 4), with no statistically significant correlation between the SL parameters and IOP measurements found at 1M, 3M, and 12M (Table 5; Figure 8).

## 4. Discussion

CLASS is a modification of NPDS, and the hypothesized IOP lowering mechanism following this procedure is complex. It has been established that IOP reduction in NPDS is achieved with a number of components: subconjunctival filtering bleb, intrascleral filtering bleb, suprachoroidal filtration, and enhanced episcleral vein outflow of aqueous from Schlemm's canal [14, 15]. As the intrascleral structures cannot be evaluated in the slit-lamp examination, our study incorporated a combination of IBAGS and AS-OCT to provide a complete assessment of the external and internal filtering bleb components in CLASS.

IBAGS and AS-OCT have been used by Oh et al. [38] to present a comparison of bleb morphology between trabeculectomy and deep sclerectomy. Three months postoperatively, on the clinical grading, their trabeculectomy group had significantly higher, broader, and less vascular blebs than the eyes after deep sclerectomy. In AS-OCT scans, trabeculectomy eyes were more likely to show microcysts and fluid beneath the scleral flap than the eyes after deep sclerectomy. For both trabeculectomy and deep sclerectomy, larger horizontal bleb extent was associated with lower IOP. In our study, IBAGS revealed significant differences in the bleb height and its evolution up to one year between the successful and failed CLASS groups. The majority of patients in the successful group presented shallow blebs up to one year (H1 rate decreasing from 91% to 57% and 52% at 1M, 3M, and 12M, resp.). Interestingly, initially there was no evidence of flat blebs in this group (0% at 1M), but 48% manifested a flat bleb at 12M. The majority of the failed group presented no bleb (H0 E0) from the early postoperative period (59%), with the rate increasing to 88% at 3M and reaching 100% at 12M. The literature reported the formation of a subconjunctival filtering bleb to be as low as 50% of NPDS cases [39], and some authors described flat blebs in 100% of the successful NPDS with collagen implant and mitomycin C patients [12]. Chihara et al. [37] reported IOP decrease in 23 of their 37 NPDS patients despite no detectable filtering bleb. Moreover, they concluded that presence of the filtering bleb did not warrant postsurgical low-teen IOP as the IOP in eyes with or without the bleb was not significantly different. All of our successful group patients showed mild vasculature (V2) from the early postoperative period and the majority (83%) maintained this score for one year. However, at 3M and 12M, the vascularity was not useful to distinguish between the successful and failed CLASS, as there was no significant difference in V1 and V2 rates between the groups. Interestingly, none of the successful patients presented V3, while this grade was assigned to all the failed group patients who eventually underwent trabeculectomy due to early CLASS failure.

Visualization of intrascleral structures following trabeculectomy and NPDS has been reported with both UBM and AS-OCT. The findings for the NPDS are contradictory in terms of IOP correlation and scleral bleb measurements. Some authors found an inverse correlation between the postoperative IOP and intrascleral lake height [12, 13, 35], anteroposterior extent [35] or volume [35], while the others conclude that the size of the lake is not correlated with degree of the IOP reduction [36, 37]. It has been postulated that the scleral lake may facilitate the fluid drainage to subconjunctival space or supraciliary space through an orifice of the Schlemm's canal, disruption of the trabecular meshwork, gaps between the scleral flap and the scleral bed, or transscleral hydraulic conductivity [37]. It has been also reported that collapse of the lake leads to poor IOP control [40]. Therefore, a range of methods to prevent the collapse has been proposed, including a collagen implant [41], a slit incision of Schlemm's canal [42], or goniopuncture [43]. In the study of Perez-Rico et al. [35], the mean follow-up from the MMC augmented NPDS to AS-OCT examination was 58 months, the open intrascleral space was found in 100% of evaluated eyes, and the IOP showed significant negative correlations with the maximum intrascleral space anteroposterior length and height. Mavrakanas et al. [12] investigated a homogenous group of 25 patients after MMC-augmented NPDS with collagen implant, and all their patients manifested clinically flat blebs, which were scanned with the Visante AS-OCT at 8 ± 5 months after the surgery. None of their studied eyes manifested subconjunctival blebs, and 17 out of 25 eyes showed microscopic conjunctival fluid collections. All eyes presented an intrascleral bleb with the mean height of 0.586 mm, and the IOP was inversely correlated with the intrascleral bleb height. Fernandez-Buenaga et al. [13] performed Visante AS-OCT in three groups of NPDS patients with different types of scleral implants. At the median of 15.5 months following the surgery, the average scleral bleb height in vertical scans was 0.63 mm. Two of their groups showed statistically significant correlation between the SL height and IOP decrease; however, these correlations were weaker than those described by Mavrakanas et al. [12].

In contrast, other studies, including ours, have reported that the size of the lake is not correlated with the degree of IOP reduction. We found a statistically significant decrease in SL anteroposterior extent and SL height over time (Tables 3 and 4), with no correlation between the above SL parameters and IOP measurements found at any time point (Table 5; Figure 8). The IOP in our successful group has remained low regardless of the time-dependent decrease in the lake size, which is consistent with the findings described by Park et al. [44]. Chihara and Hayashi [37] used the spectral domain AS-OCT and reported poor correlation between the scleral lake volume and the IOP reduction rate in their group. The lake extent was not found to be correlated with the rate of IOP reduction in the earlier UBM studies [36, 40].

Microcysts were a common finding, and we did not notice significant difference in the rates of microcysts between the successful and failed groups (Table 1). Importantly, in the failed group, AS-OCT revealed the evidence of inaccurate ablation, anterior synechia, iris incarceration, and fibrosis (Figures 7(a)–7(a)). These OCT findings were crucial to explain the mechanism of an early and late CLASS failure and decide about further intervention in the particular patients. The scleral lake was the only AS-OCT finding which we detected in all the successful patients (100%) through the whole follow-up period of the study (Table 2). The successful patients presented with high rates of TDM integrity (87–91%), IF (87–83%), and SCF (83%) (Table 2; Figures 6(a) and 6(b)). Chihara and Hayashi found the TDM integrity in 32 of 37 eyes.

Our results may suggest that the surgical technique influences bleb morphology. Similar to NPDS, in CLASS, a lamellar scleral flap is created and a layer of the local underlying sclera is removed. Area of the evaporated tissue becomes the site of the intrascleral filtering bleb, when the operation is successful. Surprisingly, we noticed that our average SL height measurements in CLASS seem to be lower, compared with the values for NPDS described by Mavrakanas et al. [12] and Fernandez-Buenaga et al. [13]. Greifner et al. [8] compared the results of CLASS with NPDS and explained that, in the CLASS procedure, the aiming beam of the CO_2_ laser is focused on the area of the Schlemm's canal and the pigmented trabeculum located underneath. The authors suggested that, in contrast to NPDS, in CLASS, there is no TDM or “window” dissected anteriorly to the clear cornea but only the thin residual membrane of pigmented trabeculum that separates the anterior chamber from the intrascleral space. Indeed, in our AS-OCT scans performed in the successful group, we found the thin membrane of residual trabeculum, which was in the close proximity to the iris root. However, the membrane was extending anteriorly towards Descemet's membrane (Figures 3, 4, 6(a), and 6(b)).

Overall, our clinical grading results indicate that the most likely slit-lamp presentation of the successful CLASS was a shallow bleb with mild vasculature, especially observed in the early postoperative period (Figure 1(a)). In the 12-month perspective, however, the bleb maintenance was not proved to be a mandatory manifestation of the successful IOP control.

Our results, including the external bleb evolution together with the AS-OCT findings, seem to support the explanation which was postulated by Chihara and Hayashi [37] for the NPDS. We draw the conclusion that the mechanism of aqueous outflow in CLASS may be the filtration from the scleral wound and the percolation to the supraciliary space, rather than the transscleral percolation to the subconjunctival space, especially in the long-term postoperative observation. In the late postoperative period, the subconjunctival bleb may be a sign of the effective filtration when it matches the AS-OCT findings of residual trabeculo-Descemet's membrane integrity, maintained scleral lake, and intrascleral fluid (Figures 1(c) and 6(a)). Among our failed group patients, three months postoperatively, there were only two cases of the shallow blebs (H1E2) and no blebs were recorded at 12 months in this group. However, in the early period (1M), the bleb formation was observed in 41%, which may suggest that the subconjunctival fluid collection in the failed group could be a consequence of a short-term diffuse leakage around the surgical site. Therefore, the subconjunctival bleb observed in the early postoperative period does not seem to be a useful predictor of a long-term CLASS success. The role of the transscleral outflow has been discussed in the literature. Some authors postulated [37] that, if this mechanism was important to reduce IOP, eyes with a large lake would have a large area for the fluid percolation and would be expected to have a greater level of IOP reduction. Other authors emphasized that, as the hydrostatic pressure of the supraciliary space is 0.8–4.7 mmHg lower than in the anterior chamber, the pressure gradient may be a driving force and the lake is presumably an aqueous reservoir for uveoscleral outflow [45]. Moreover, the UBM findings of pooled supraciliary aqueous after viscocanalostomy support the theory for increased uveoscleral outflow following the nonpenetrating procedures [46].

Our AS-OCT findings may be a valuable contribution to a discussion on the limitations of nonpenetrating and penetrating procedures in glaucoma surgery. On one hand, the mechanism of IOP control following CLASS seems to be less dependent on the external bleb formation. Therefore, the procedure may potentially overcome the challenges in bleb maintenance, which remain a common concern in trabeculectomy. On the other hand, our results prove that CLASS still carries a risk of iris incarceration and fibrosis (Figure 7), which may result in early and late failure of both penetrating and nonpenetrating surgery. We refer an interested reader to a thorough elaboration on the results, complications, and limitations of the both types of glaucoma procedures in [47], as that matter is out of the scope of this article. The literature [47] provides a detailed discussion on the safety and efficacy profile of CLASS compared with trabeculectomy.

In conclusion, this is the first report on the aspects of external and intrascleral surgical sites in CLASS. As the nonvolumetric AS-OCT examination is safe and easy to perform, its analysis might provide a meaningful insight for postoperative follow-up in CLASS. The main limitation of our study was the lack of bleb wall reflectivity measurement and access to the JPG files only. We strongly support the postulate of the other authors [12, 13, 37] that a validated approach to OCT pixel intensity measurement is required. Such analysis could be performed either by automatic software provided by the manufacturer or by raw data files after processing. We believe that the objective bleb reflectivity assessment with the AS-OCT is essential for the future studies, as it could provide more information on the wound healing process and enhance our ability to detect early signs of fibrosis and glaucoma surgery failure.

## Figures and Tables

**Table 1:** Comparison of bleb morphology in the successful and failed groups using the Indiana Bleb Appearance Grading Scale (IBAGS) at 1 month (1M), 3 months (3M), and 12 months (12M).

	1M					3M					12M				
	Successful (*N*=23)		Failed (*N*=17)		*P*	Successful (*N*=23)		Failed (*N*=17)		*P*	Successful (*N*=23)		Failed (*N*=10)		*P*
	*n*	%	*n*	%		*n*	%	n	%		*n*	%	*n*	%	
H0	0	0	10	59	<0.001^∗∗^	10	43	15	88	0.01^∗^	11	48	10	100	0.005^∗∗^
H1	21	91	7	41	0.002^∗^	13	57	2	12	0.01^∗^	12	52	0	0	0.005^∗∗^
H2	2	9	0	0	0.499^∗∗^	0	0	0	0	—	0	0	—	0	—
E0	2	9	10	59	0.002^∗^	10	43	15	88	0.01^∗^	11	48	10	100	0.005^∗∗^
E1	1	4	7	41	0.006^∗∗^	2	9	2	12	1^∗∗^	2	9	0	0	1^∗∗^
E2	19	83	0	0	<0.001^∗^	11	48	0	0	0.001^∗∗^	10	43	0	0	0.015^∗∗^
V1	0	0	0	0	—	4	17	0	0	0.123^∗∗^	4	17	0	0	0.289^∗∗^
V2	23	100	12	71	0.009^∗∗^	19	83	12	71	0.456^∗∗^	19	83	10	100	0.289^∗∗^
V3	0	0	5	29	0.009^∗∗^	0	0	5	29	0.009^∗∗^	0	0	0	0	—
S0	23	100	16	94	0.425^∗∗^	23	100	17	100	—	23	100	10	100	—
S1	0	0	1	6	0.425^∗∗^	0	0	0	0	—	0	0	0	0	—

^∗^Chi-squared test; ^∗∗^Fisher's exact test (due to low expected values).

**Table 2:** AS-OCT findings in the successful and failed groups at 1 month (1M), 3 months (3M), and 12 months (12M).

	1M					3M					12M				
	Successful (*N*=23)		Failed (*N*=17)		*P*	Successful (*N*=23)		Failed (*N*=17)		*P*	Successful (*N*=23)		Failed (*N*=10)		*P*
	*n*	%	*n*	%		*n*	%	n	%		*n*	%	*n*	%	
Scleral lake (SL)	23	100	5	29	<0.001	23	100	0	0	<0.001	23	100	0	0	<0.001
TDM integrity	20	87	2	12	<0.001	21	91	0	0	<0.001	21	91	0	0	<0.001
IF	20	87	2	12	<0.001	19	83	2	12	<0.001	19	83	1	10	<0.001
SCF	19	83	7	41	0.017	19	83	4	24	0.001	19	83	4	40	0.035
Microcysts	16	70	13	76	0.73^∗∗^	18	78	13	76	1^∗∗^	18	78	7	70	0.673

^∗^Chi-squared test. ^∗∗^Fisher's exact test (due to low expected values).

**Table 3:** AS-OCT scleral lake anteroposterior extent measurements over time in the successful group.

Time	SL anteroposterior extent (*μ*m)								*P* ^∗^
	*N*	Average	SD	Median	Minimum	Maximum	Q1	Q3	
1M	23	1466.26	374.29	1359	896	2489	1264	1657.5	0.003
3M	23	1407.7	398.99	1299	875	2422	1149.5	1665	
12M	23	1293.74	361.53	1233	832	2010	999.5	1543	12M < 1M, 3M

^∗^Repeated measures ANOVA + post hoc analysis (paired Student's *t*-tests with Bonferroni correction).

**Table 4:** AS-OCT scleral lake height measurements over time in the successful group.

Time	SL height (*μ*m)								*P* ^∗^
	*N*	Average	SD	Median	Minimum	Maximum	Q1	Q3	
1M	23	430.13	123.39	421	199	634	333.5	532	<0.001
3M	23	399.87	130.87	341	250	652	292.5	501.5	
12M	23	344.7	130.84	273	205	590	239	449.5	12M < 1M, 3M

^∗^Friedman test + post hoc analysis (paired Wilcoxon tests with Bonferroni correction).

**Table 5:** Correlation between AS-OCT scleral lake measurements (SL anteroposterior extent, SL height) and intraocular pressure (IOP) in 23 patients presenting clinically successful CLASS at 1 month (1M), 3 months (3M) and 12 months (12M) following the surgery. The correlation coefficient (r) is not significant.

Scleral lake measurement	Correlation with IOP					
	1M		3M		12M	
	/r/	*P* ^∗^	/r/	*P* ^∗^	/r/	*P* ^∗^
SL anteroposterior extent (*μ*m)	0.149	0.496	0.121	0.582	−0.051	0.818
SL height (*μ*m)	0.022	0.921	0.051	0.817	−0.05	0.82

^∗^Spearman rank correlation coefficient.

**Table 6:** Demographics and baseline characteristics.

CLASS (*n*=66)		*n* (%)
Sex	Male	30 (45)
	Female	36 (55)
Glaucoma type	POAG	34 (52)
	XFG	32 (48)
	Median (quartiles)	
Age	71 (64–77)	
IOP (mmHg)	23 (22–25)	
Drugs	3 (3-4)	

**Table 7:** Complete and qualified success rates over time.

Time	Complete success		Qualified success	
	*n*	%	*n*	%
After a month (1M)	48	73	51	77
After 3 months (3M)	33	50	51	77
After 12 months (12M)	23	35	49	74
